# Controllable Plasmonic Nanostructures induced by Dual-wavelength Femtosecond Laser Irradiation

**DOI:** 10.1038/s41598-017-16374-6

**Published:** 2017-12-11

**Authors:** Weina Han, Lan Jiang, Xiaowei Li, Qingsong Wang, Shaojun Wang, Jie Hu, Yongfeng Lu

**Affiliations:** 1 Beijing Engineering Research Center of Applied Laser Technology, Institute of Laser Engineering, Beijing University of Technology, Beijing, 100124 P.R. China; 2 Laser Micro/Nano Fabrication Laboratory, School of Mechanical Engineering, Beijing Institute of Technology, Beijing, 100081 P.R. China; 3 Laser Micro/Nano-Fabrication Laboratory, Department of Mechanical Engineering, Tsinghua University, Beijing, 100084 P.R. China; 4 Department of Electrical and Computer Engineering, University of Nebraska-Lincoln, Lincoln, NE 68588-0511 USA

## Abstract

We demonstrated an abnormal double-peak (annular shaped) energy deposition through dual-wavelength synthesis of the fundamental frequency (*ω*) and the second-harmonic frequency (2*ω*) of a femtosecond (fs) Ti:sapphire laser. The annular shaped distribution of the dual-wavelength fs laser was confirmed through real beam shape detection. This uniquely simple and flexible technique enables the formation of functional plasmonic nanostructures. We applied this double-peak fs-laser-induced dewetting effect to the controlled fabrication and precise deposition of Au nanostructures, by using a simple, lithography-free, and single-step process. In this process, the double-peak energy-shaped fs laser pulse induces surface patterning of a thin film followed by nanoscale hydrodynamic instability, which is highly controllable under specific irradiation conditions. Nanostructure morphology (shape, size, and distribution) modulation can be achieved by adjusting the laser irradiation parameters, and the subsequent ion-beam polishing enables further dimensional reduction and removal of the surrounding film. The unique optical properties of the resulting nanostructure are highly sensitive to the shape and size of the nanostructure. In contrast to a nanoparticle, the resonance-scattering spectrum of an Au nanobump exhibites two resonance peaks. These suggest that the dual-wavelength fs laser-based dewetting of thin films can be an effective means for the scalable manufacturing of patterned-functional nanostructures.

## Introduction

Plasmonic nanostructures have recently attracted considerable academic attention, with demonstrations of extraordinary optical transmission^1–3^, perfect lensing^4–6^, magnetic response at visible wavelengths^7^, and potential for developing new optical elements^8,9^. Metallic nanostructures are efficient systems for electromagnetic field control at the nanoscale level because of their ability to support resonantly excited collective electron oscillations, known as localized surface plasmons^10,11^. Metallic nanostructures can be considered as the basic elements of complex structures and metamaterials; they exhibit desirable responses to electromagnetic fields based on their structures and arrangements^12,13^.

Functional plasmonic nanostructures can be achieved using various methods including direct ion-beam milling^14^ and multistage e-beam and nanoimprint lithography techniques^15,16^. However, additional fabrication steps are required for building substrate templates and film patterns so that the nanostructures can be ordered into a functional nanostructure. Moreover, these approaches are extremely expensive and time-consuming when fabricating close-packed arrays of plasmonic nanostructures. Therefore, lithography-free and single-step methods are desirable for ordered or disordered large-scale nanostructuring. Chemical synthesis^17–19^ and monodisperse nanoparticle colloidal methods^20^, termed single-step techniques, are suitable for large-area fabrication of disordered nanostructure arrays but require additional steps for isolated nanostructures to be arranged into highly ordered arrays. During the last decade, direct laser nanostructuring^21–23^ of metal films has been demonstrated to be a promising alternative approach; nanoparticles can be fabricated through the “green” and high-throughput laser-induced transfer of material from a thin film placed a certain distance from a receiving substrate^22,23^. Meanwhile, spatial pulse shaping has been applied to the manipulation of subwavelength nanostructures by directly ablation on the surface of bulk materials. Such as the silicon nanostructure on the bulk silicon surface can be fabricated by the fs vortex pulses^24,25^.

Among the various manufabrication approaches that have been proposed in previous studies, dewetting of thin films into nanostructures for scalable nanomanufacturing of various materials is one of the most prevalent methods for functional plasmonic nanostructure fabrication. Laser radiation^26–29^, ion-beams^30^, and thermal heating^31–33^ have been used as thermal sources for initiating a spontaneous dewetting process. Nevertheless, traditional uncontrolled, spontaneous dewetting yields random arrangements of nanostructures with well-known instability. Controlled fabrication of ordered metal nanostructure arrays can be achieved only through an additional lithography process^27,28,30,31,34^, which is expensive and time-consuming. Thus, the development of a lithography-free, single-step dewetting method for well-ordered nanostructure fabrication is required for a broad range of plasmonic applications.

Recently, Makarov *et al*. demonstrated plasmonic nanostructure fabrication by using a femtosecond laser-induced dewetting process with gentle material removal by multiple (approximately 10^4^) vertical laser pulse scanning^35^. However, this method requires a highly precise processing platform for the narrow period of laser scanning; moreover, the high number of incident laser pulses restricts the processing efficiency. In the present study, we demonstrated the use of direct, single-shot exposure of a thin metal film to a tightly focused, dual-wavelength fs laser pulse for dewetting of a thin gold (Au) film on fused silica and Si substrates. The experimental results demonstrated that an annular irradiated structure can be fabricated by single-shot, dual-wavelength fs laser pulse irradiation with a relatively large focusing spot. Through shrinkage of the focal spot, a concentric circular-shaped fabricated structure was transformed into a nanoscale Au dot at the center of the beam spot. The shape and size of the fabricated nanostructures were determined according to the pulse energy and metal film thickness. Furthermore, we reduced the sizes of the fabricated nanostructures by subsequent isotropic ion-beam polishing and produced isolated plasmonic nanostructures on a dielectric/semiconductor substrate. The single-step, lithography-free, dual-wavelength fs-laser-induced dewetted nanostructures were arranged into periodic and complex structures by a high speed scanning method that involved laser frequency and scanning speed. The plasmonic properties of the nanostructures were measured through dark-field (DF) microspectroscopy. Numerical simulation was conducted in the framework of finite-difference time-domain calculations of electromagnetic near fields. This method provides a promising pathway for the high-efficiency fabrication of metal nanostructures for plasmonic applications.

## Results and Discussion

### Dual-Wavelength fs Laser Printing of a Concentric Micropatch

Compared with a long laser pulse, a fs laser pulse fundamentally changes the laser-material interaction mechanism in some aspects. That leads fs laser-material interaction a complex process, especially for the dual-wavelength fs laser pulses. Several studies have reported ablation based on dual-colorirradiation. Okoshi *et al*. proved that dual-color fs pulses can result in deeper and faster etching of polyethylene. During the ablation, an isolated carbon, in addition to C = O and C = C-H bonds, was formed on the surface after treating 2ω or dual-color pulses. The higher photon energy of 2ω pulses cuts the chemical bonds of the polyethylene to form a modified layer on the ablated surface^36^. Moreover, the relative phase of the synthesized dual-wavelength waveform plays an important role in the ablation process. The ablation of PMMA using a dual-color waveform was demonstrated with a dependence on a multiphoton ionization rate related to the relative phase of the dual-wavelength waveform^37^. However, few studies have focused on chromatic aberrations due to the dispersion of the phase accumulated by light during propagation. For materials with normal dispersion, refractive lenses have larger focal distances for red light than for blue light. Therefore, the dual wavelengths are spatially separated by the focusing lens. Focusing with aberrations at different wavelengths (see Fig. S1(a) in Supplementary Information) and different focal spots (see Fig. S1(b) in Supplementary Information) could be observed at different focal planes (see Fig. S2 in Supplementary Information for the far-field intensity distribution focusing by the refractive lens simulated by finite-difference time-domain (FDTD)). In our study, we measured the real beam shape by passing a Gaussian-shaped laser beam through abarium borate (BBO) crystal and focusing it using a lens with a focal length of 200 mm. The experimental results revealed an annular intensity distribution (see Fig. S3 in Supplementary Information). Moreover, the real beam shape at different wavelengths on the focal plane within the Rayleigh range was analyzed. A Gaussian-shaped distribution can be observed for the incident fs laser pulses with wavelengths of 800 and 400 nm (see Fig. S4 in Supplementary Information). Thus, we propose that the focusing aberration leads to the beam reshaping by the dual-wavelength synthesis with a double-peak intensity distribution.

**Figure 1:**
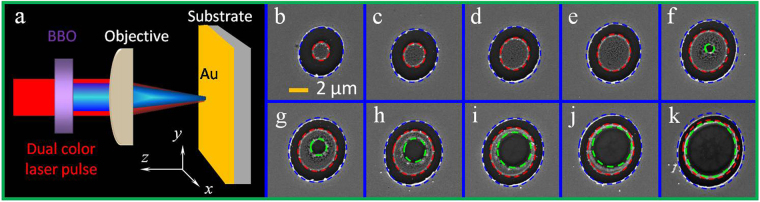
(**a**) Schematic of the experimental setup. (**b**–**k**) SEM images of ablated structures imprinted by a single pulse of a dual-wavelength fs laser. The pulse fluence increased from 0.01 to 0.055 μJ with a step length of 0.005 μJ. All of the images share the same scale bar.

The geometrical morphology of the ablated surface structures can imprint the deposited pulse energy of a focused laser pulse with a large focal spot. In the experiments, the dual-wavelength fs laser pulse was first focused using a 4× magnification microscope (Daheng Optics, NA = 0.1) with a relatively large focal spot. The ablated surface structures imprinted by single laser pulses on Au films were studied to demonstrate the abnormal intensity distribution of the dual-wavelength fs laser. When the pulse energy was adjusted from 0.01 to 0.055 μJ, regular concentric circular microstructures were observed. The changes in the concentric circular microstructures observed at different laser pulse energies are illustated in Fig. 1. At low pulse energy (Fig. 1b–e), a Au micropatch perforated by sloppy nanoholes (marked by the dotted red lines) was observed at the central spot. The sloppy nanoholes can be attributed to the heat effect. As shown in the figure, the entire ablated area (marked by dotted blue lines) increased with the pulse energy, similar to the area of the micropatch. At higher pulse energies, the sloppy nanoholes on the micropatch structures were ablated, forming another characteristic feature, namely a circular ablated structure, as shown in the area marked by green dotted lines in Fig. 1f–k. Apparently, the area of ablated circular structure (marked by the green line) increased as the pulse energy was increased for the increasingly deposited pulse fluence. When the pulse energy exceeded 0.06 μJ, the micropatch with sloppy nanoholes (area between the red and green line) vanished (not shown here), and a circular ablated microstructure was formed, similar to the structures formed when conventional Gaussian-shaped laser pulses are used. Thus, the experimental results also demonstrated an annular energy deposition shaped by the dual-wavelength fs laser pulses.

The isolated wavelengths (800 nm/400 nm) are compared with the fixed pulse energy. The results revealed that the single laser pulse with a wavelength of 400 nm demonstrated no ablation or modification (see Fig. S5a in Supplementary Information). A circular ablation of the Au film was observed under single laser pulse irradiation with a wavelength of 800 nm (see Fig. S5b in Supplementary Information). Studies revealed that an enhancement of absorption/reflection was confirmed in fused silica by dual-wavelength fs laser pulses at zero delay for the creation of defect states or free electron plasma^38^. However, the ablation mechanism of metallic thin films is different from those the solid bulk materials^39^, and more detailed experimental and theoretical analysis is required to investigate the detailed mechanisms.

### Directed Dewetting by a Double-Peak Energy fs Laser Pulse

Furthermore, in this study, we demonstrated that the dual-wavelength fs laser can be used to fabricate functional plasmonic nanostructures by decreasing the focal spot size. We experimented with the directed dewetting of thin Au films on SiO_2_ and Si substrates by using a dual-wavelength fs laser pulse for the fabrication of Au nanostructures. These experiments involved laser-induced dewetting of thin metal films by using tightly focused, dual-wavelength fs laser pulses with an annular intensity distribution. The single dual-wavelength fs laser pulse irradiation method was employed to fabricate the Au nanostructure array, simply and efficiently, by using a high-speed laser scanning method that involved the laser frequency and the scanning speed. The periods in the orthogonal directions can be controlled by the laser scanning speed and the applied laser frequency, or by the scanning step, which is determined by the scanning mode (scanning along horizontal or vertical directions).

**Figure 2:**
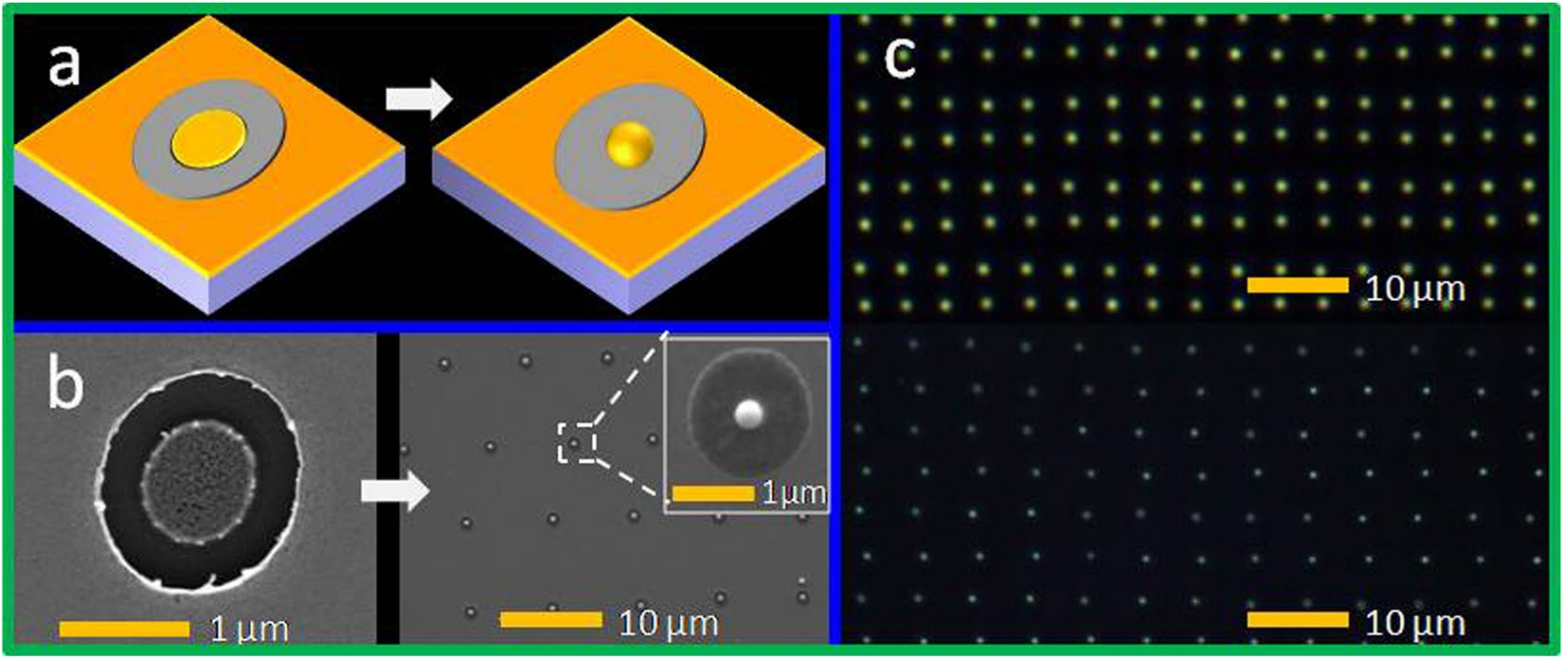
(**a**) Schematic of single nanoparticle formation under dual-wavelength fs laser irradiation from a Au film on a dielectric/semiconductor substrate. (**b**) SEM images of the concentric structures and Au nanoparticles on Si substrate fabricated by dual-wavelength fs laser irradiation of a 30-nm Au film at pulse energies of 0.012 and 0.024 μJ. (**c**) Array of Au nanostructures fabricated by this method and visualized with DF microscopy; the pulse energies were 0.021 μJ and 0.026 μJ for the upper panel and lower panel, respectively.

The nanostructuring of the metal film on the substrate by a single dual-wavelength fs laser pulse was divided into two physical stages. As mentioned above, concentric micropatches (Fig. 1b–e) can be fabricated by single dual-wavelength fs laser irradiation with a relatively large focal spot size (×4 magnification microscope objective) with a size larger than the uncertainty of the grooved edges (area between the blue dashed line and red dashed line shown in Fig. 1b). The circular patch at the center of the spot was thermally isolated because the thermal conductivities of the SiO_2_ substrate and air were two and four orders of magnitude smaller than that of Au, respectively. Therefore, the isolated circular patch was easily heated to the temperature at which the film underwent the dewetting process. The dewetting temperature was markedly lower than the melting point, 1337 K, of the bulk Au material^40^. Pressure relaxation is in the order of picoseconds for metals; for subpicosecond laser pulses, with pulse durations shorter than the time of the pressure relaxation, the laser heating was followed by high-pressure wave excitation^41^, which disturbed the surrounding area and may have decreased the dewetting temperature^42^. According to previous studies, a single particle can be formed from a single patch when the patch width-to-height ratio is smaller than a certain value^32^. Thus, the heated circular Au patch can be transformed into a nanostructure during the dewetting process by reducing the size of the central micropatch, as shown in Fig. 2a,b.

The decreased size of the central micropatch can be obtained by using the objective with higher magnification. Here, we used a microscope objective (Newport) with ×20 magnification and a NA of 0.45 to reduce the circular micropatch in the center of the irradiated spot. During illumination of the thin metal film by a single, tightly focused dual-color fs laser pulse, absorption in the top metal layer led to ultrafast heating and local melting of this layer. The oxide and semiconductor substrates (the melting temperatures of which were considerably higher) remained unaffected by these laser pulse energies. The melting of Au leads a volume reduction. This technique enabled high-quality, direct writing of Au nanostructures and an arbitrary arrangement of nanostructures at a high processing rate. An example of dewetting a large-area array of Au nanostructures produced from a 40-nm-thick Au film with different diameters and a periodicity of 4 μm is shown in Fig. 2c as a DF microscopic image. And the pulse energies were 0.021 and 0.026 μJ for the upper panel and lower panel, respectively. The laser scanning method involved the laser frequency and scanning speed enabled the fabrication of large-area Au nanostructures irradiated by single laser pulse at an extremely high production rate; the speed of this technique depends on the laser scanning rate, which is usually determined by the processing platform (2000 μm/s for our platform; 100 s/mm^2^ for the scanning speed of 2000 μm/s with a periodicity of 5 μm). Arbitrary two-dimensional (2D) arrangements of Au nanostructures were successfully fabricated on 20–40-nm Au films over a large area (see Fig. S6 in the Supplementary Information). This method can be applied to glass substrates and to semiconductor substrates (see Fig. S7 in the Supplementary Information).

**Figure 3:**
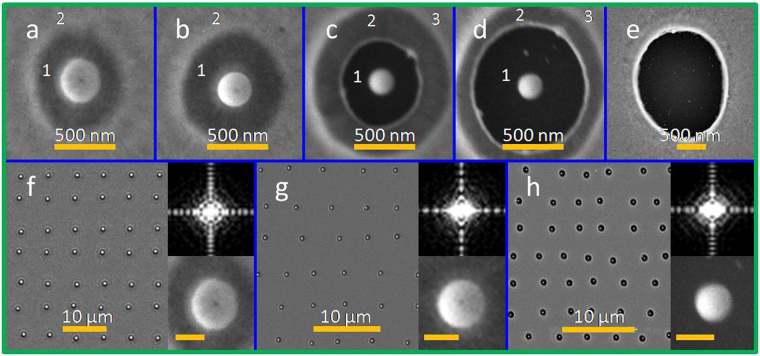
(**a**–**e**) SEM images of Au nanostructures fabricated from a 40-nm film on a SiO_2_ substrate at various pulse energy levels: a 0.020 μJ, b 0.022 μJ, c 0.025 μJ, d 0.027 μJ, e 0.032 μJ. f–h SEM images of large-area Au nanodisc, nanodome, and nanoparticle arrays corresponding to the nanostructures shown in a, b, and d. Right lower insets: enlarged SEM images of typical nanostructures from the arrays (scale bar, 200 nm). Right upper insets: Fourier spectra of the single nanostructure SEM images.

The size and morphology of the Au nanostructures can be varied by adjusting the laser pulse energy (with a range of 0.020–0.030 μJ for 40-nm-thick films and within a range of 0.008–0.013 μJ for 20-nm-thick films). Different scenarios can be observed depending on the quantity of laser pulse energy on the metal surface. Figure 3a–e show SEM images of Au nanostructures fabricated from a 40-nm Au film as a sequence of increased pulse energy. Four main regimes were observed: (I) circular molten nanodisc from the melting Au film (Fig. 3a); (II) transition from a circular nanopatch to a single nanodome (Fig. 3b); (III) single Au nanoparticle surrounded by a peripheral rim (Fig. 3c,d); and (IV) formation of a nanoring (Fig. 3e).

Notably, because of surface tension, the molten region in the central area formed a molten Au nanodisc, as shown in Fig. 3a. When the laser pulse energy was increased, the melting circular nanopatch was pulled up toward the optical center by thermocapillary forces; a semispherical nanobump was thus formed (Fig. 3b). This process was characterized by the onset of strong thermocapillary forces and corresponding microscopic hydrodynamic flows (Marangoni convectional flow)^39^.

Several typical areas can be defined in different regimes by changing the pulse energy. As can be seen in Fig. 3a,b, thinned Au film (marked as Area 1) can be observed between the molten nanopatch or nanobump and the Au film (marked as Area 2). With a further increase in the pulse energy, the central nanobump was cut and separated from the metal film; it contracted into a single nanoparticle accompanied by a peripheral rim, as shown in Fig. 3c,d. In this regime, the nanoparticle was isolated by the substrate (marked by Area 1), the thinned Au film (marked by Area 2), and the originally deposited Au film (marked as Area 3). (For details of the material characterization, see Fig. S8 in Supplementary Information). Furthermore, the diameter of the peripheral rim increased with the pulse energy. Area 1is smaller in Fig. 3c than in Fig. 3d. The size of Area 1 can be made equal to the size of Area 2 by controlling the pulse energy. At higher pulse energies, the markedly thinned metal film (Area 2in Fig. 3c,d) and condensed nanoparticle shell broke up, forming a microhole through the film with a pronounced molten ring (Fig. 3e). The diameters of the microholes increased gradually with the pulse energy. The peripheral rims in different regimes were also produced by the same mechanism; although in other regimes, the height increases were mild because the molten Au spread outwards radially.

### Manipulation of Single Au Nanostructure

**Figure 4:**
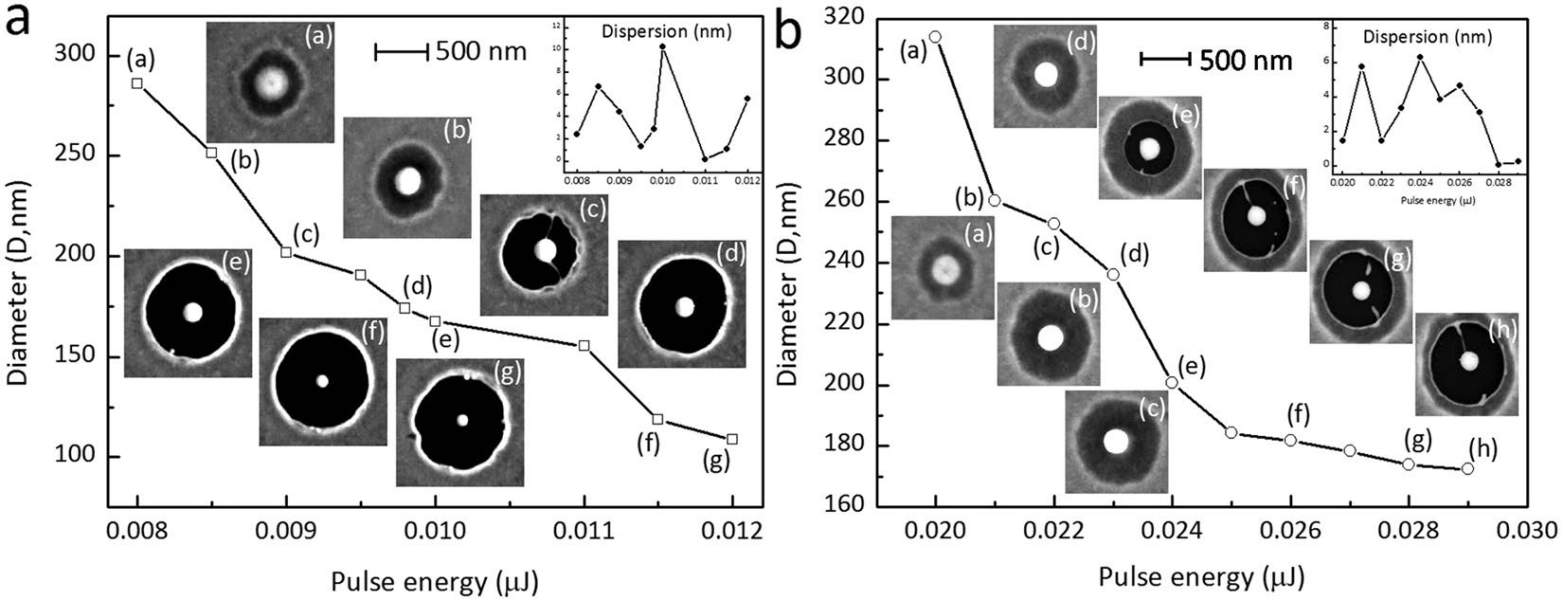
Effects of pulse energy on the diameter of Au nanostructures from a 40-nm-thick Au film and b 20-nm-thick Au film. The irradiation pulse energy range for the nanostructures ranged from 0.020 to 0.029 μJ for the 40-nm-thick film and from 0.008 to 0.012 μJ for the 20-nm-thick film. The insets show SEM images of Au nanostructures produced at different pulse energies; all of the images share the same scale bar.

The experimental results showed that the sizes of Au nanostructures produced with a fixed film thickness can be varied by adjusting the pulse energy. Figure 4 reveals the effect of the pulse energy on the diameter of the produced Au nanostructure. The insets show SEM images of the Au nanostructures produced from (Fig. 4a) 40-nm-thick and (Fig. 4b) 20-nm-thick Au films with different laser pulses. For the 40-nm-thick Au film, as shown in Fig. 4a, the diameters of the Au nanostructures ranged from 310 to 170 nm at energy pulse levels ranging from 0.020 to 0.029 μJ. The size of the produced nanostructure was substantially smaller than the beam size (approximately 2.4 μm without the BBO crystal at the 1/*e* ^2^ beam radius of the input beam). With a thinner initial film, a similar evolution was observed in the nanostructure morphology. Notably, even smaller nanoparticles were produced. For example, with the 20-nm-thick Au film, when the pulse energy ranged from 0.008 to 0.012 μJ, the diameters of the Au nanostructures ranged from 290 to 100 nm, as shown in Fig. 4b. For Au nanostructure production, the required pulse energy decreased as the film thickness decreased. Furthermore, the experimental results revealed that the Au nanostructures could not be reproduced for a film thickness of 10 nm. As mentioned, transformation of the central circular patch into a single nanostructure can be achieved, which is due to the circular patches being too small in comparison to the typical wavelength of Rayleigh-type instability^32^. Therefore, films that are too thick and those that have large patches can hardly be heated homogeneously during laser irradiation; thus, controllable dewetting cannot be achieved for such films.

### Ion Beam-Assisted Polishing Enabled Size Reduction of Au Nanostructures

During the dual-wavelength fs laser pulse-induced dewetting process, the molten Au at the centers formed different morphologies based on the pulse energy deposited, and the molten rims formed nanoring structures. Such a modified structure not only has a visible front surface but also a hidden rear surface, which can be revealed by removing the remainder of the deposited thin film.

**Figure 5:**
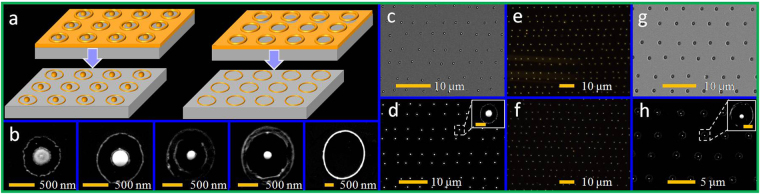
(**a**) Schematics of the ion-beam-assisted polishing process. (**b**) SEM images of Au nanostructures corresponding to Fig. 3a–e after the isotropic etching process. SEM images of Au nanodomes fabricated from a 40-nm-thick film on a SiO_2_ substrate, before c and after d isotropic etching polishing; the pulse energy was 0.022 μJ. (**e**,**f**) DF field microscopy images corresponding to c and d, respectively. SEM images of Au nanoparticles fabricated from a 40-nm-thick film on a SiO_2_ substrate before g and after h isotropic etching polishing; the pulse energy was 0.028 μJ Inset in h shows the isolated double nanoring–nanoparticle composite structure.

Because of the marked height increases of the dewetted Au nanostructures during the morphological transformation, the initial thin films were removed through etching. This enabled the formation of various plasmonic nanostructures, such as nanoring structures and composite nanodisc–nanoring structures, nanodome–nanoring structures, and nanoparticle–nanoring structures, as shown in Fig. 5a. Furthermore, the molten peripheral rim was removed because it was thinner than the Au nanostructure.

After removal of the thin film, pure Au nanostructures remained on the substrate. In this study, the sample was etched through isotropic ion-beam polishing, and the flat initial film area was gradually thinned and removed. Figure 5b shows the polished Au nanostructures which correspond to those in Fig. 3a–e. As shown in Fig. 5b, the influence of the ion beam resulted in the formation of different isolated nanostructures at specific places on the sample surface, which had previously been irradiated by a single dual-wavelength fs laser pulse. For Regime I (nanopatch) and Regime II (nanodome), a nanoring-nanostructure (nanopatch/nanodome) composite structure was formed. The nanoring formation was based on the molten rim, as shown in Fig. 3a,b. For Regime III, two molten rims were formed (the rims of Areas 1 and 2 marked in Fig. 3c,d) by the irradiation of a dual-wavelength fs laser pulse, resulting in the formation of a nanoparticle with two nanorings around it (as shown in Fig. 5c,d); that is, an isolated double nanoring–nanoparticle was formed. Furthermore, the size of the inner nanoring increased with the pulse fluence, which was identical to the aforementioned molten rim evolution in Regime III. The width of the formed nanorings, based on the molten rims in Regimes I–III, was approximately 26 ± 5 nm. For Regime IV, a microhole structure, an isolated single nanoring, was formed after ion-beam polishing. The width of the nanoring was approximately 80 nm. Moreover, in each case, from Regimes I to IV, the experimental results demonstrated appropriate reproducibility sufficient for large-scale nanostructure array fabrication with different morphologies. Figure 5d and Fig. 5h show the SEM images of large-area 2D arrays of the ion-beam polished, isolated nanoring–nanodomestructures and double nanoring–nanoparticle structures, which correspond to the unpolished structures shown in Fig. 5c and Fig. 5g, respectively. Figure 5e,f show the DF images corresponding to Fig. 5c,d, namely, unpolished structures and polished structures, respectively. (More 2D arrays of Au nanostructures can be seen in Fig. S9 in Supplementary Information.) Furthermore, the sizes of the Au nanostructures were precisely reduced by deliberately controlling the etching time.

### Optical Characterization

**Figure 6:**
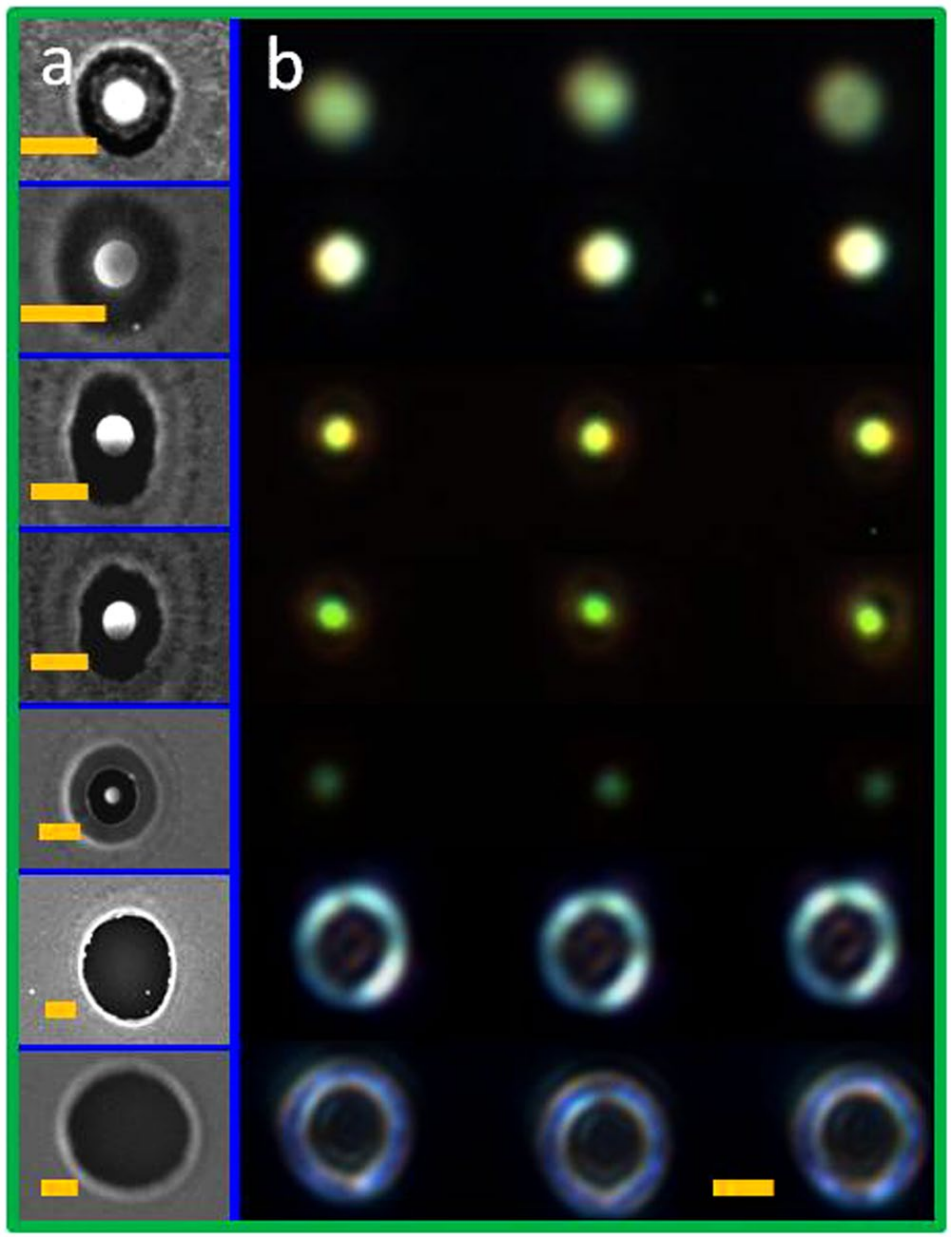
(**a**) SEM images (scale bar, 500 nm) of nanostructures fabricated from 40-nm-thick Au film at slightly different laser pulse energies of 0.021, 0.022, 0.023, 0.024, 0.025, 0.032, and 0.034 μJ from top to bottom in each line. (**b**) Changes in the optical responses of the nanostructures are visualized by DF microscopic images (scale bar, 5 μm).

As mentioned, the sizes of the Au nanostructures were manipulated by adjusting the laser pulse energy. SEM images of the nanostructures obtained with different pulse energies on a 40-nm-thick film are shown in Fig. 6a. The diameters of the nanopatches, nanodomes, and nanoparticles were adjusted to be within the range of 170–320 nm, and their DF microscopic images are given in Fig. 6b.

Spectrum reshaping of the far field radiation patterns for the Au nanoring structures was observed. Detailed AFM detection revealed no structural changes inside the nanoring. According to previous studies, annular-shaped nanostructures—as a special type of cavity—are particularly attractive and exhibit some fascinating phenomena such as the focusing effect^43,44^ and dark multipolar plasmon excitation^45–47^. Because they possess these properties, annular-shaped nanostructures are considered highly practicable in areas such as biosensors, color filters, nanoantennas, and negative indices of refraction^47–52^. The scattering images of the nanorings in Fig. 6b reveal evident far field radiation patterns with annular morphology appeared inside the nanorings. When nanorings with larger diameters were used, the fringes increased from one ring to two rings. This reshaping effect can be interpreted in the context of the plasmonic lens effect of the metallic annular-shaped single nanowire^43^.

**Figure 7:**
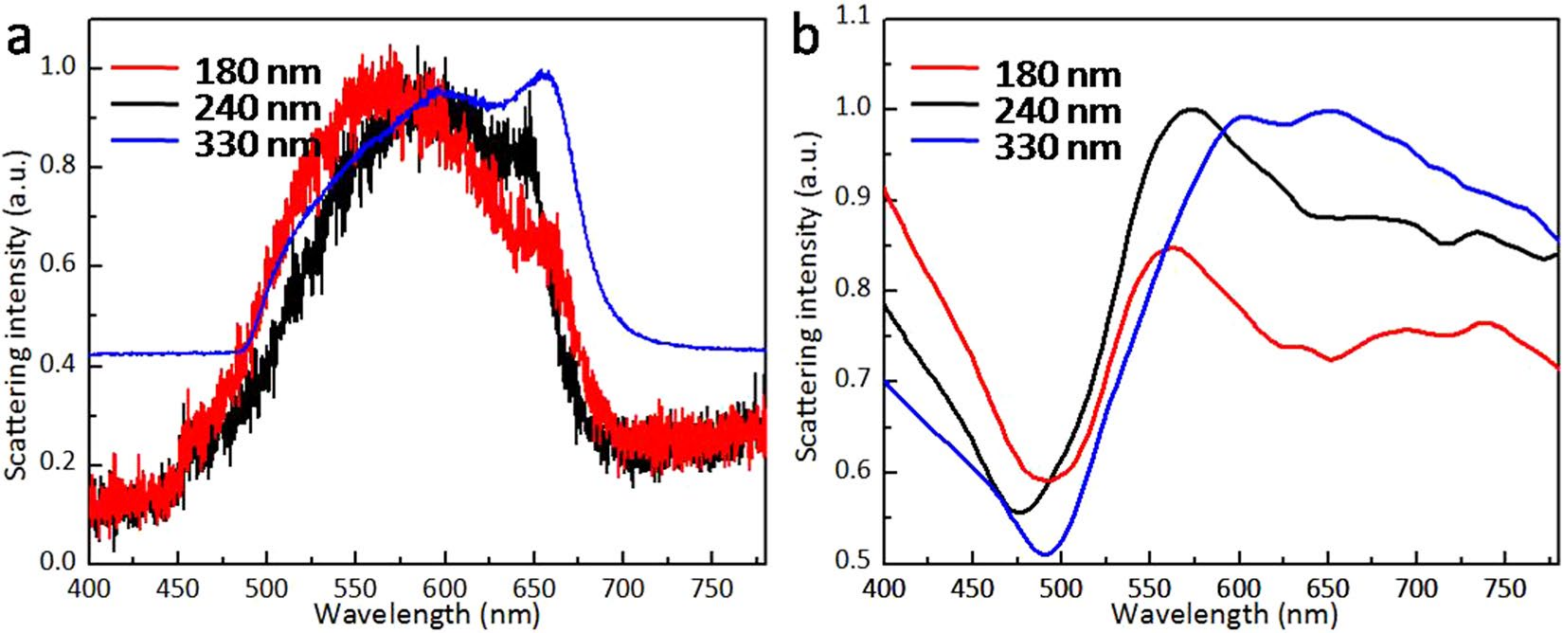
(**a**) Normalized DF back-scattering spectra measured from individual Au nanostructures with diameters of 330 nm (blue curve), 180 nm (red curve), and 240 nm (black curve), obliquely illuminated (incidence angle of 70°) by a nonpolarized white-light source. (**b**) Simulated scattering cross sections for Au nanostructures with diameters of 330 nm (blue curve), 180 nm (red curve), and 240 nm (black curve), irradiated by a propagating plane light wave.

The collective electron oscillations resonantly excited by the noble metal nanostructures is the main element for plasmonic metasurfaces and filters^53–55^. The resonant oscillations are called localized surface plasmon resonance (LSPR). Studies show that the frequency of LSPR strongly depends on the size, shape, and environment surrounding the nanostructures. In this study, the scattering spectra of Au nanostructures with different morphologies deposited on a glass substrate directly after dual-wavelength fs laser irradiation are shown in Fig. 7a. Two resonance peaks were observed for both nanodomes (330 nm) and nanoparticles (180 nm and 240 nm). The approximately 300-nm Regime II nanodomes had nearly-spherical shapes and were partially embedded into the substrate, because of dewetting on the molten substrate. As shown in Fig. 7a, the scattering spectra from an ordered 330-nm nanodome array (blue curve) revealed two resonances at *λ* ≈ 600 and *λ* ≈ 650 nm (for the measurement details, see *Methods*). Numerical simulations based on FDTD (for the simulation details, see Fig. S10 in Supplementary Information) were performed for the Au nanodome with a diameter of 330 nm and 250 nm height (see Fig. S11 in Supplementary Information for the detailed profiles). As shown in Fig. 7b (blue curve), the calculated scattering section shows good agreement with the experimentally observed optical properties. This mode corresponds to a high-order multipole resonance, which is qualitative agreement with previous studies^56^. A large nanostructure size may decrease the effective wavelength of the incident light, leading to the excitation of higher-order multipole resonance. Compared with ordered monodisperse nanodomes, the approximately 180-nm fabricated nanoparticles in Regimes III and IV exhibited an evident broad resonance at *λ* ≈ 550 nm; a weak resonance peak at *λ* ≈ 650 nm was also observed, as shown in Fig. 7a (red curve). Meanwhile, for a nanoparticle with a diameter of 240 nm, the resonance exhibited a blue shift at an evident resonance at 575 nm and a weak resonance peak at 625 nm, as shown in Fig. 7a (black curve). These resonances were in favorable qualitative agreement with our numerical simulation (Fig. 7b) for the nanoparticle array of 180-nm (red curve in Fig. 7b) and 240-nm (black curve in Fig. 7b) Au spheres embedded in a fused silica substrate. The weak resonance peak may have been induced by the spherical nanoring structure. This observed peak corresponds to the electric dipole resonance. Meanwhile, the experimental and simulated results confirmed a blue-shifted resonance with decreased morphological size. The observed far-field resonant scattering light can be attributed to the excitation of the different localized plasmon modes. According to the Mie Theory, the increased size of the nanopaticles leads to the changes in the light-scattering properties. As the size increased, the scattering spectral profiles change the shift toward longer wavelengths due to increasing contributions to the usual electric dipole contribution^57^.

### Fabrication Flexibility

**Figure 8:**
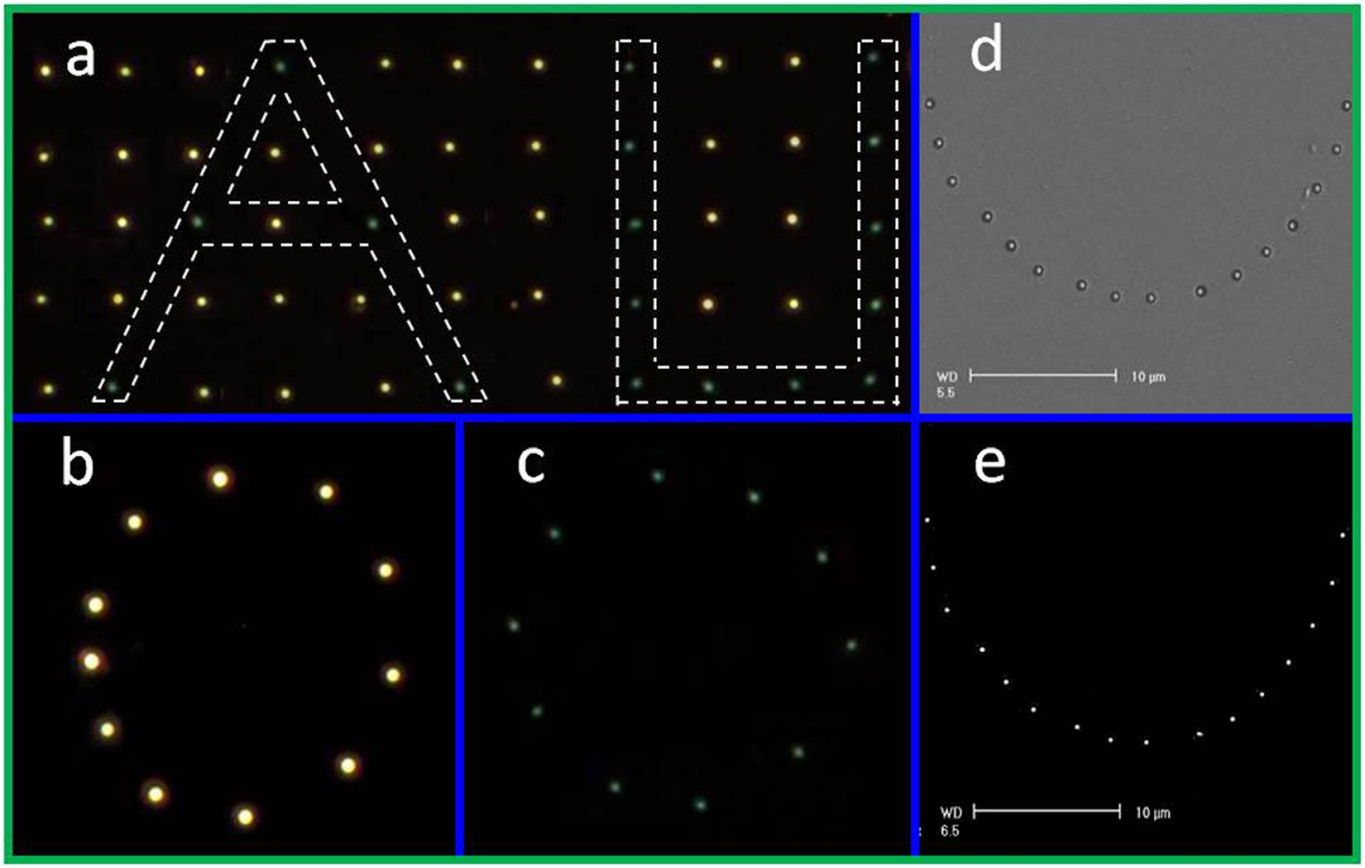
(**a**–**c**) DF microscopic images of Au nanoparticles of various sizes fabricated from a 40-nm-thick film on SiO_2_ substrates. The nanoparticles within the white dotted lines in a were fabricated with a laser energy of 0.026 μJ; the others were fabricated at 0.021 μJ. The pulse energies were 0.023 and 0.024 μJ in b and c, respectively. (**d**,**e**) SEM images of Au nanodomes with a semicircular arrangement fabricated on a 30-nm-thick film on a fused silica substrate before d and after e isotropic ion-beam etching. The pulse energy was 0.016 μJ.

This single-step, mask-free, laser scanning method involving laser frequency and scanning speed makes the fabrication a flexible process. The arrangements and morphologies of these Au nanostructures can be artificially programmed. This process makes the generation of Au nanostructures with adjustable optical properties possible. DF microscopic images of the results of the selective processing of single nanostructures fabricated from a film with a thickness of 40 nm are shown in Fig. 8a (see Fig. S12 in Supplementary Information for the schematic). The nanostructures outside the dotted white lines are nanodomes in Regime II, which were fabricated at a pulse energy of 0.021 μJ. The nanostructures within the dotted white lines are nanoparticles in Regime III. They were fabricated at a pulse energy of 0.026 μJ, producing a visible color change. An arbitrary arrangement can be achieved (see Fig. S13 in Supplementary Information for more arrangements). Figure 8b,c shows the DF microscopic images of nanodomes b and nanoparticles c with a circular arrangement. The pulse energies were 0.023 and 0.024 μJ, respectively. Figure 8d shows a semicircular arrangement of nanodomes formed with a pulse energy of 0.016 μJ, and the corresponding polishing result is shown in Fig. 8e. More complex patterns with specific morphologies can be achieved through predefined programmable platform control.

## Conclusion

The proposed novel single-step, lithography-free method, based on dual-wavelength fs-laser-directed dewetting with double-peaked (annular shaped) energy deposition on a sample surface for direct Au nanostructure fabrication, reveals new possibilities for creating high-quality, arbitrary arrangements of plasmonic nanostructures with different morphologies on a large scale. The laser scanning method for single dual-wavelength fs laser pulse irradiation involved the laser frequency and scanning speed enables fabrication at relatively high rates with well-ordered arrays. Use of high repetition rate lasers (in the megahertz regime) and faster translation stages can substantially enhance the processing rate even further. Moreover, this method can be applied to flexible materials such as dielectric or semiconductor substrates, allowing precise control of their microscopic properties. The experimental and numerical optical analysis revealed this method’s functional resonant absorption for plasmonic applications. With isotropic etching, isolated Au nanostructures with different morphologies can be placed on substrates. By adjusting the etching time, the sizes of nanostructures can be reduced without destroying the substrates. The combination of dual-wavelength fs laser dewetting and subsequent isotropic etching constitutes a straightforward method for scalable nanofabrication. We believe that the proposed method is a new stage in the rapidly growing field of large-area nanophotonics. This method can be useful for metal coloration as well as high-performance and cheap printing of functional ordered substrates for plasmonics, field emission devices, solar cells, biosensing, and metamaterials etc.

## Methods

### Fabrication

The experimental setup is shown schematically in Fig. 1a. A commercial Ti:sapphire laser regenerative amplifier system was used, which provided 50 fs laser pulses at a central wavelength of 800 nm (*λ*_1_). To realize the dual-wavelength waveform, the frequency ω was doubled in a 100-μm-thick, Type І beta barium borate (*β*-BBO) crystal in front of the microscope objective to generate 2ω pulses at 400 nm (*λ*_2_). The dual-wavelength fs laser pulse was focused by the objective [4 × (NA = 0.1)/20 × (NA = 0.45)] on the surface of the Au film. Through adjustment of the incident angle, the intensity ratio *E*_*2ω*_/*E*_*ω*_ was controlled to a maximum value of approximately 30%, which is the maximum conversion efficiency of the BBO crystal. Neutral density filters were employed to enable variable adjustment of the laser intensity. Several Au thin films, with thicknesses in the range of *h* = 20–40 nm, were sputtered onto optically smooth bulk SiO_2_ glass or Si substrates with adhesion layers of 3 nm thick Cr film. Each sample was placed on a computer-controlled, six-axis moving stage with a positioning accuracy of 1 μm in the x and y directions and 0.5 μm in the *z* direction.

### Isotropic Ion-Beam Polishing

The nanostructures fabricated on the thin Au film through single-shot fs laser pulse irradiation were then etched using a normally incident unfocused Ar+ ion beam to a depth equal to the thickness of the thin film by using a commercial apparatus. The isotropic metal etching reduced the sizes of the Au nanostructures while maintaining its morphology simply by controlling the etching time. A single nanoparticle and a nanoring of reduced size were achieved simultaneously.

### Characterizations

The morphology characterizations of the structured samples were characterized through scanning electron microscopy (SEM). The Fourier spectra of SEM images were obtained using Image-Pro® Plus software. Optical characterization of the single Au nanostructure fabricated was performed by an optical fiber spectrometer, which was connected to a DF microscope setup. A DF condenser (M, NA = 0.9) was used to illuminate the Au nanostructures. The light scattered by the individual nanostructures was imaged using a microscope objective with ×100 magnification and an NA of 0.55 simultaneously on a charge-coupled device (CCD) camera; the irradiation was performed at an incidence angle of 70° to the surface normal; scattered signals were collected using an objective Mitutoyo M Plan APO NIR (NA = 0.7).

### Numerical Simulations

The finite-difference time domain (FDTD) was used to simulate the scattering cross-section of the fabricated nanostructures. The simulation domain included a structure with perfectly matched layers in all directions. The Au dielectric function was obtained from the experimental data of Johnson and Christy based on the three measurements of reflection and transmission at normal incidence, together with p-polarized transmission at an angle of 60° ^58^, and the refractive index of SiO_2_ was *n* = 1.45. Total-field scattered-field source at given wavelength ranging from 400 to 800 nm was used to irradiate the single Au nanostructures (see Fig. S10 in the Supplementary Information for the detailed modeling geometry of the corresponding nanostructures). The size of the square unit cell was set as 2 × 2 × 2 nm^3^, and the computational volume was limited by perfectly matched layers.

## Electronic supplementary material

Supplementary Information
