# Supplementary material for: Controllable Plasmonic Nanostructures induced by Dual-wavelength Femtosecond Laser Irradiation
Source: Sci Rep. 2017 Dec 11;7:17333. doi: 10.1038/s41598-017-16374-6 (PMC5725531; doi:10.1038/s41598-017-16374-6)
Supplement: Supplementary file 1 — Supplementary Information [file 41598_2017_16374_MOESM1_ESM.doc]

**Supplementary Information**

**Controllable Plasmonic Nanostructures induced by Dual-wavelength Femtosecond Laser Irradiation**

Weina Han^1,2^, Lan Jiang^1,*^, Xiaowei Li^1^, Qingsong Wang^1^, Shaojun Wang^1^, Jie Hu^1^, Yongfeng Lu^3^

^1^Beijing Engineering Research Center of Applied Laser Technology, Institute of Laser Engineering, Beijing University of Technology, Beijing, 100124, PR China

^2^Laser Micro/Nano Fabrication Laboratory, School of Mechanical Engineering, Beijing Institute of Technology, Beijing, 100081, PR China

^3^Department of Electrical and Computer Engineering, University of Nebraska-Lincoln, Lincoln, NE 68588-0511, USA.

**Characterization of the Dual-Color Laser after Objective Focusing**

Refractive optics relies on the phase gradually accumulated through propagation. For material with normal dispersion, refractive lenses have larger focal distances for red light than for blue. In most transparent materials in the visible, the refractive index *n* decreases with increasing wavelength (normal dispersion). Because the focal lens *f* is inversely proportional to *n* - 1, the resulting effect of the dual color (800 nm + 400 nm) laser focusing by the lens is shown in Fig. S1. Therefore, a spatial separation of the dual wavelengths is formed by the focusing lens. At different focal plans (A, B and C), focusing with aberrations at different wavelengths could be observed. And different focal spots can be found at different focal plans, as shown in Fig. S1. Recently, Aieta *et al.* reported multiwavelength achromatic metasurfaces by dispersive phase compensation. ^S1^

**Figure S1.** a, The schematic diagram of the blue (400 nm) and red (800 nm) light focusing by refractive lens. b, Schematic diagram of the focal spots at different focal planes corresponding to that in a.

The focusing of the laser beam by the lens is demonstrated with finite-difference time-domain (FDTD) simulations. Focusing with aberrations with different wavelengths can be observed. The simulated results with wavelength of 800 nm are shown in Fig. S2. The far-field intensity distribution for the laser beam with wavelength of 800 nm is shown in Fig. S2(a). As shown, we observe good focusing around 45 μm for wavelength of 800 nm (plane A-A marked by the red dashed line in Fig. S2(a)). Fig. S2(b) shows the cross section (beyond the focusing plane defined by the Rayleigh range) across the plane B-B in (a). Double-peak intensity distribution can be found in plane B-B.

**Figure S2**. a Far-field intensity distribution for laser beam with wavelength of 800 nm. b, Cross section across the B-B plane (28 μm, marked by the blue dashed line in a) in a for wavelength of 800 m, which is beyond the focal plane define by the Rayleigh range. The red dashed line A-A in a indicts the focusing plane.

**Characterization of the Dual-Color Laser**

For directed dewetting, the double peak (annular shaped) energy deposition on the sample surface under dual-color fs laser irradiation is the main element in this manufacturing approach. Based on the aforementioned ablated concentric microstructures, we deduced a donut-shaped cross-sectional profile by the dual-color fs laser. As for our study in this manuscript, we conducted some experiments to investigate the detailed laser profile of the dual-color fs laser. We checked the real beam shape after the Gaussian shaped laser beam was beamed through the BBO crystal and focused by a lens with a focusing length of 200 mm. As shown in Fig. S3, the dual-color fs laser exhibited an annular-shaped intensity distribution on the focal plane within the Rayleigh range. And the focus point is confirmed by the maximum pulse intensity and minimize spot size. ^[S2]^

**
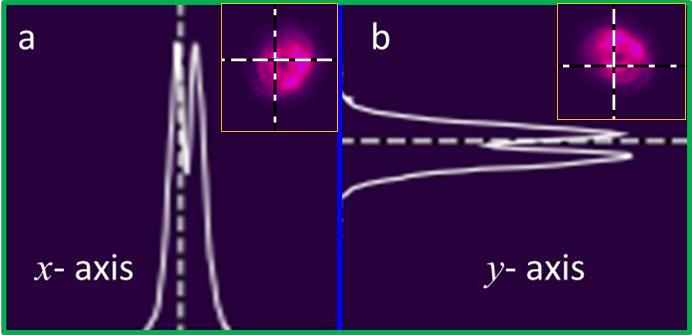
**

**Figure S3.** Real beam shape after frequency doubled by BBO crystal and focused by a lens with a focusing length of 200 nm. a, pulse energy distribution along *x*-axis; b, pulse energy distribution along *y*-axis.

The real beam shape of the fundamental frequency fs laser pulse (800 nm) focused by a lens (f = 200 nm) was checked by using the Laser Beam Profiler (Beam Gage). A Gaussian shaped intensity distribution was observed on the focal plane within Rayleigh range, as shown in Fig. S4a. Meanwhile, the extremely weak intensity of the frequency doubled fs laser pulse (400 nm) cannot be sensed by the Laser Beam Profiler, thus the CCD camera was used to check its real beam shape. The real beam shape of the frequency doubled fs laser also presents a Gaussian-shaped distribution (Fig. S4b).

**Figure S4.** a, The real beam shape on the focal spot within Rayleigh range of the fundamental frequency fs laser pulse (800 nm). b, The real beam shape on the focal spot within Rayleigh range of the frequency doubled fs laser pulse (400 nm). The incident fs laser pulses were focused by a lens (f = 200 nm).

**Figure S5.** SEM images of the irradiated area on the 40 nm Au film deposited on a SiO_2_ substrate by single fs laser pulse with wavelength of 400 nm (a) and 800 nm (b), respectively. The pulse energy of the incident fs laser pulse with wavelength of 800 nm was 0.026 μJ.

Au Nanostructure Array Formation with Different Metal Film Thickness


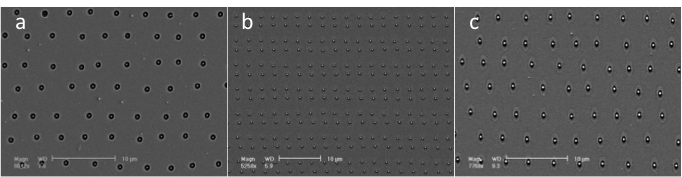


**Figure S6.** SEM images of 2D array of Au nanostructures fabricated with different Au film thickness a 20 nm; b 30 nm; and c 40 nm. The laser energies were 0.01 J, 0.015 J, and 0.025 J, respectively.

Large-Area Fabrication on Si Substrates


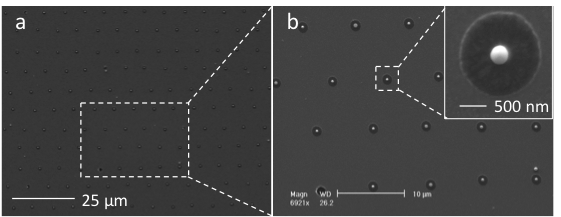


**Figure S7.** a-b SEM images of Au nanostructures fabricated from 20-nm film on Si (111) substrate with pulse fluence of 0.012 μJ. The insert in b shows a SEM image of a single Au nanodome in this array.

EDX Detection of the Irradiated Structures in Different Areas

To verify the detailed material characterization of the nanostructures in different regimes, we performed the EDX detection. For the nanodome regime (or the nanadisk regime), the EDX detection of the area 1 result is shown in Fig. S8a, it confirms the complete ablation of the Au film. The EDX detection on the area 2 with thinned Au film is shown in Fig. S8b, it confirms the existence of Au. Furthermore, the EDX detection of the initial sample surface (area 3) with 40 nm Au film deposited on the Si substrate is shown as a comparison in Fig. S8c.

**Figure S8.** EDX detection of the Au nanostructure at different regimes.

2D Array of Single Au Nanostructure Achieved by Ion Beam-Assisted Polishing


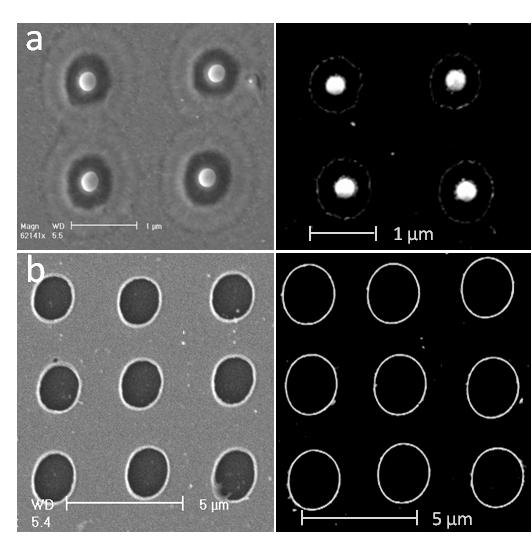


**Figure S9.** 2D array of single Au nanodome and nanoring structure.

**Numerically Calculated Distributions of the Total Normalized Electric Field Intensity around Spherical Au Particles based on FDTD Simulation**

**Figure S10.** The details of the FDTD simulations of the nanoparticle regime (*d_1,2_*, *h_1,2_*) and nanodome regime (*D*, *H*, *t*).

**Figure S11.** AFM profile of single nanostructure with diameter aroung a, 180 nm; b, 240 nm; and c, 330 nm.

**Figure S12.** Schematic of the selective fabrication process.

**Figure S13.** DF images of of Au nanodome a,b and nanoparticle c,d with different arrangement. The red arrow is provide to assist the reader in the arrange directions of Au nanostructures.

**References**

1. Aieta, F., Kats, M.A., Genevet, P., Capasso, F. Multiwavelength achromatic metasurfaces by dispersive phase compensation. *Science* **347**(6228), 1342-1345 (2015).
2. Bonse, J., Baudach, S., Krüger, J., Kautek, W., and Lenzner, M. Femtosecond laser ablation of silicon–modification thresholds and morphology. *Appl. Phys. A* **74**(1), 19-25 (2002).
